# Expression levels of a gene signature in hiPSC associated with lung adenocarcinoma stem cells and its capability in eliciting specific antitumor immune‐response in a humanized mice model

**DOI:** 10.1111/1759-7714.13440

**Published:** 2020-04-20

**Authors:** Jingbo Wang, Lijuan Shao, Liujing Wu, Wei Ma, Yuanyuan Zheng, Chaofeng Hu, Furong Li

**Affiliations:** ^1^ Translational Medicine Collaborative Innovation Center, The Second Clinical Medical College (Shenzhen People's Hospital) Jinan University Shenzhen China; ^2^ Shenzhen key laboratory of stem cell research and clinical transformation Shenzhen China; ^3^ Integrated Chinese and Western Medicine Postdoctoral research station Jinan University Guangzhou China

**Keywords:** Cancer stem cell, hiPSC, humanize mice model, immune response, tumor vaccine

## Abstract

**Background:**

Previous studies have reported that cancer stem cells (CSCs) play a key role in tumorigenesis, metastasis, and recurrence. CSC‐based vaccination confers better protection in tumor cells. However, isolation and cultivation of CSCs are difficult. This study aimed to explore the similarities between CSCs and induced pluripotent stem cells (iPSCs).

**Methods:**

ALDH1+ cancer stem cells were isolated from lung adenocarcinoma patients and their gene expression patterns compared with human induced pluripotent stem cells (hiPSCs). In addition, a tumor vaccine was developed using hiPSC and unmethylated cytosine‐guanine (CpG). Finally, the antitumor properties of the vaccine were evaluated in a humanized mouse model.

**Results:**

Preimmunization of iPSC+CpG elicited stronger antigen presentation and cytotoxic T cell response which suppressed the growth of tumors. Adoptive transfer of spleen T cells from the vaccine preimmunized mice inhibited tumor growth in unvaccinated recipients without any side effects.

**Conclusions:**

This study suggests a universal strategy for tumor therapy which simplifies future clinical procedures. Therefore, the application of hiPSC elicits tumor protective responses.

## Introduction

Cancer is among the leading causes of death globally with increased incidence rate. Tumor cells display marked heterogeneity in cellular morphology, proliferation activity, genetic lesions as well as therapeutic responses.^1^ However, their molecular mechanisms remain unclear and this makes it difficult to develop universal therapeutic strategies against cancer. In 1838, Johannes Müller a German pathologist demonstrated the stem cell origin of tumors.^2^ Further, the cancer stem cell (CSC) theory proposes that tumor growth is driven by a small population of stem cells.^3^ It is believed that CSCs are responsible for tumor recurrence as well as chemoresistance.^4^ Various studies have been carried out to explore the origin of CSCs and some have reported that they originate from differentiated cells through a “pathological nuclear reprograming” process,^5^, ^6^, ^7^ which is similar to that of induced pluripotent stem cells (iPSCs).

Takahashi *et al*. discovered iPSCs,^8^, ^9^ whereby the reprogramming process was carried out to maintain both the proliferation and differentiation potential of the stem cells. Transcription profiles of good quality iPSC and embryonic stem cells are nearly identical.^10^ RNA sequencing also revealed that human and murine iPSCs express tumor associated antigens.^11^ According to a study carried out by Ohnishi *et al*. premature termination of the reprogramming process led to cancer development as the reprogrammed pluripotent stem cells acquired oncogenic phenotypes.^12^ This partially explains the origin and development of CSCs. In addition, several CSC‐related metabolic features have been discovered in iPSCs which is an indication of the similarities between these cells.^6^

Tumor vaccination is an emerging field in cancer therapy and tumor antigens are widely used.^13^ The variability of tumor‐specific antigens makes the identification of new tumor antigens a difficult process.^14^, ^15^ Therefore, the application of whole‐cell vaccines provides an alternative in which the vaccine is directed against a broad range of antigens.^16^ Immunization with embryonic materials to generate antitumor responses has been developed for some time, but the lack of suitable materials and ethical challenges limits its application.^17^, ^18^, ^19^ Previous studies have proved that CSC‐based vaccines elicit more effective antitumor responses compared with unselected tumor cells.^20^, ^21^ However, the isolation and identification of CSCs are challenging.

Based on the similarities between CSCs and iPSCs, vaccination with iPSCs may provide a representative panel of antigens similar to those elicited by CSCs. The application of iPSC to induce antitumor immunity has been previously explored in mice models.^11^, ^17^, ^22^ In this study, we explored the possibility that human fibroblast‐derived iPSC could function as a tumor vaccine and provide protective immune responses in a humanized mouse model. Moreover, multiple cancer vaccines benefit from a combination adjuvant approach and immunostimulatory molecules which activate Toll‐like receptors (TLR) through pathogen‐associated molecular patterns.^23^, ^24^ Unmethylated cytosine‐guanine (CpG) motif‐containing oligodeoxynucleotides (ODNs) are potential TLR9 agonists which can directly activate cytokine secretion, DC maturation and antigen presentation, thereby enhancing vaccine specific cellular and humoral responses.^25^ It has been widely used in cancer vaccines during both preclinical and clinical studies.^26^, ^27^ Therefore, we used CpG ODNs as an adjuvant combined with iPSC to enhance the antitumor immunity of the iPSC‐based vaccines.

## Methods

### Cells and animal models

B‐NDG (NOD‐Prkdcscid Il12rgtm1/Bcgen) mice at 5–6 weeks of age were purchased from Beijing Biocytogen Co., Ltd. (Beijing, China) and maintained under specific pathogen‐free conditions. All mice were exposed to total body Co60 irradiation before stem cell transplantation. Humanized mouse models were established using human umbilical cord CD34+ cells which were injected intravenously. Fluorescence‐activated cell sorting (FACS) was used to detect the percentage of human CD45+ cells after six weeks and mice with a percentage higher than 20 were used for further experiments. All animal experiments were approved by the ethics committee board and animal care committee of Jinan University and performed according to the institutional guidelines.

Human fibroblast‐derived iPSC was purchased from Guangzhou Institutes of Biomedicine and Health, Chinese Academy of Sciences (Guangzhou, China) and cultured in mTeSR 1 medium (Stem Cell Technologies, USA). The lung tumor cancer cell lines A549 and H1975 were cultured in DMEM medium with 10% FBS under normal culture conditions.

### Isolation of lung adenocarcinoma stem cells

Tissue samples of primary tumors were collected from lung adenocarcinoma cancer patients who had undergone surgical resection at the department of thoracic surgery, Shenzhen people's hospital. The following criteria were used to select patients for inclusion into the study: (i) no other previous treatment including surgery, neoadjuvant chemotherapy, irradiation, etc; (ii) no further metastasis and known malignancy; and (iii) pathologically diagnosed and histologically confirmed lung adenocarcinoma cancer. All patients received written informed consent before sample collection.

The tissues were rinsed thrice with PBS, mechanically sliced into small pieces and the Primary Tumor Cell Isolation Kit (IMMORTECH, China) used to isolate the primary tumor cells. All the samples were passed through 70 μM nylon cell strainers (Facon, BD, USA) and centrifuged for five minutes at 300 *g*. RBC lysis buffer (Santa Cruz Biotechnology, TX, USA) was used to remove the red blood cells. Finally, the ALDEFLOUR assay kit (Stemcell Technologies, British Columbia, Canada) was used to isolate ALDH^high^ CSCs from the isolated tumor cells according to the manufacturer's protocol. As a negative control, an aliquot of the cell samples was treated with diethylaminobenzaldehyde, a special ALDH inhibitor. The ALDH+ cells treated with activated ALDEFLOURTM reagent were analyzed using a FACS cell sorter (Sony, Japan).

### Identification of isolated CSCs

The following monoclonal antibodies were used to identify the stem cells: APC mouse IgG1, κIsotype control; FITC mouse IgG1, κIsotype control; APC mouse IgG1, κIsotype control; anti‐human CD24‐FITC; anti‐human CD34‐APC; anti‐human CD44‐APC; anti‐human CD45‐APC; anti‐human CD90‐FITC; anti‐human CD106‐FITC; anti‐human CD133‐APC; anti‐humanABCG2‐PE and Alexa flour 488 OCT4 (all from Biolegend, USA). Cells were stained and analyzed by flow cytometry.

Colony formation assay was used to test the ability of the cell to form colonies. Single‐cell suspensions were prepared at a concentration of 2000 cells/well in tissue culture plates pretreated with Matrigel in DMEM (supplemented with 1% penicillin‐streptomycin and 0.1% FBS) at 37°C, 5% CO_2_. Daily replacement of the culture media was done for10 days, after which the cell monolayer was fixed and stained using a 500 μL crystal violet staining solution and incubated at room temperature for 10 minutes. The solutions were carefully aspirated and the cell monolayer washed several times until the plates were clear. The culture plates were dried at room temperature and colonies consisting of >50 cells were counted.

Sorted ALDH1‐cells and ALDH1+cells at 1000 cells per well were suspended in 150 μL PBS‐Matrigel (1:1) and subcutaneously injected into parallel sites (ALDH‐ in the left flank and ALDH+ in the right flank) of four to seven week old female Balb/c nude mice. After the mice had been injected, tumor formation was recorded and their bodyweight was measured and recorded every two days.

### Gene expression data analysis

Total RNA of the isolated CSCs was extracted using trizol reagent and RNA sequencing performed using the Illumina HiSeq X10 platform. RNA sequencing data of the human fibroblast‐derived iPSC were downloaded from the GEO database (https://www.ncbi.nlm.nih.gov/geo/query/acc.cgi?acc=GSM3097434). The data were processed as described by Ittai Ben‐Porath *et al*.^2^

### Vaccine preparation, immunization and serum collection

For each mouse, 2 × 10^6^ human iPSCs were collected and washed with sterile PBS three times, followed by 6000 rads irradiation. CpG (Invivogen, San Diego, USA) was dissolved in PBS to a final concentration of 5 μM. Mice were anesthetized with 2% isoflurane (RWD, Shenzhen, China) and were subcutaneously injected with 100 μL of PBS, iPSC, CpG, and iPSC+CpG each week for four weeks. The injection sites were changed every week. Serum samples were collected at two and four weeks following the first immunization.

### IgG binding assay

A549 and H1975 cells were collected, washed with PBS three times and resuspended in 100 μL PBS with the addition of 2 μL of serum from the vaccinated mice and incubated at 4°C for 30 minutes. Following this, the cells were then washed three times, resuspended in 100 μL PBS and incubated with an anti‐IgG FITC secondary antibody (ThermoFisher Scientific, USA) at 4°C for 20 minutes. The cells were analyzed using a flow cytometer (BD Bioscience, CA, USA). Data were analyzed using FlowJo software (Three Star, CA, USA).

### Organ histopathology

The heart, liver, and kidneys explanted from vaccinated mice were processed for histopathology. Briefly, the organs were fixed overnight in 4% paraformaldehyde and transferred to 70% ethanol for another 24 hours. Fixed samples were embedded in paraffin and 5 μm sections were cut and stained with hematoxylin and eosin (H&E) for histological analysis.

### Immune cell differentiation assay

The mice were sacrificed and the blood and splenocytes were isolated and resuspended in 100 μL PBS. FcR‐blocking reagent (BD PharMingen, San Diego, USA) was used to reduce nonspecific binding of antibodies. To detect antigen‐presenting capacity, cells were divided in two tubes where one was incubated with anti‐CD11c PE, anti‐HLA FITC and anti‐CD80 APC antibodies (all from Biolegend, San Diego, USA) for 20 minutes on ice and a second tube was incubated with anti‐CD11b PE and anti‐CD80 APC antibodies (Biolegend). Cytotoxic T cell detection was performed by staining the cells with anti‐CD8 PE (Biolegend) and anti‐GranzymeB FITC (eBioscience, USA) antibodies. Regulatory T cell detection was performed by staining the cells with anti‐CD4 Flu647, anti‐CD25 PE and anti‐Foxp3 FITC antibodies (Biolegend). Blood T cell differentiation was performed by staining the cells with anti‐CD4 FITC, anti‐CD44 APC, anti‐CD8 PE antibodies (Biolegend). True‐Nuclear Transcription Factor Buffer Set (Biolegend) was used for intracellular transcription factors staining. Briefly, cells were incubated with 1 mL fixing buffer for 20 minutes on ice and further incubation in the perm buffer for 30 minutes. Antibodies were diluted in perm buffer and incubated with the cells on ice for 30 minutes.

### Tumor‐bearing mice model

A total of 5 × 10^6^ A549 or H1975 cells were collected and resuspended in 100 μL PBS. A hind flank injection was performed on each mouse to observe tumor growth. Tumors were monitored and measured over four weeks. The mice were sacrificed, the tumors harvested and the final measurements collected.

### Adoptive transfer of spleen T cells

PBS, CpG and iPSC+CpG vaccinated mice were sacrificed and the splenocytes isolated. The cells were incubated with CD3 microbeads (Miltenyi, Germany) at 4°C for 30 minutes. After passing them through LS column (Miltenyi, Germany), CD3 + T cells were collected and washed with PBS. The cells were then dissolved in 100 μL PBS solution and intravenously injected into a lung tumor‐bearing mouse in the tail vein.

## Statistical analysis

The statistical analysis was performed using a two‐sided Student's *t*‐test for paired/unpaired data. GraphPad Prism software (Chicago, USA) was used to perform statistical analysis. *P*‐values <0.05 were considered to be statistically significant.

## Results

### Similar gene expression sets between CSC and iPSC

**Figure 1:**
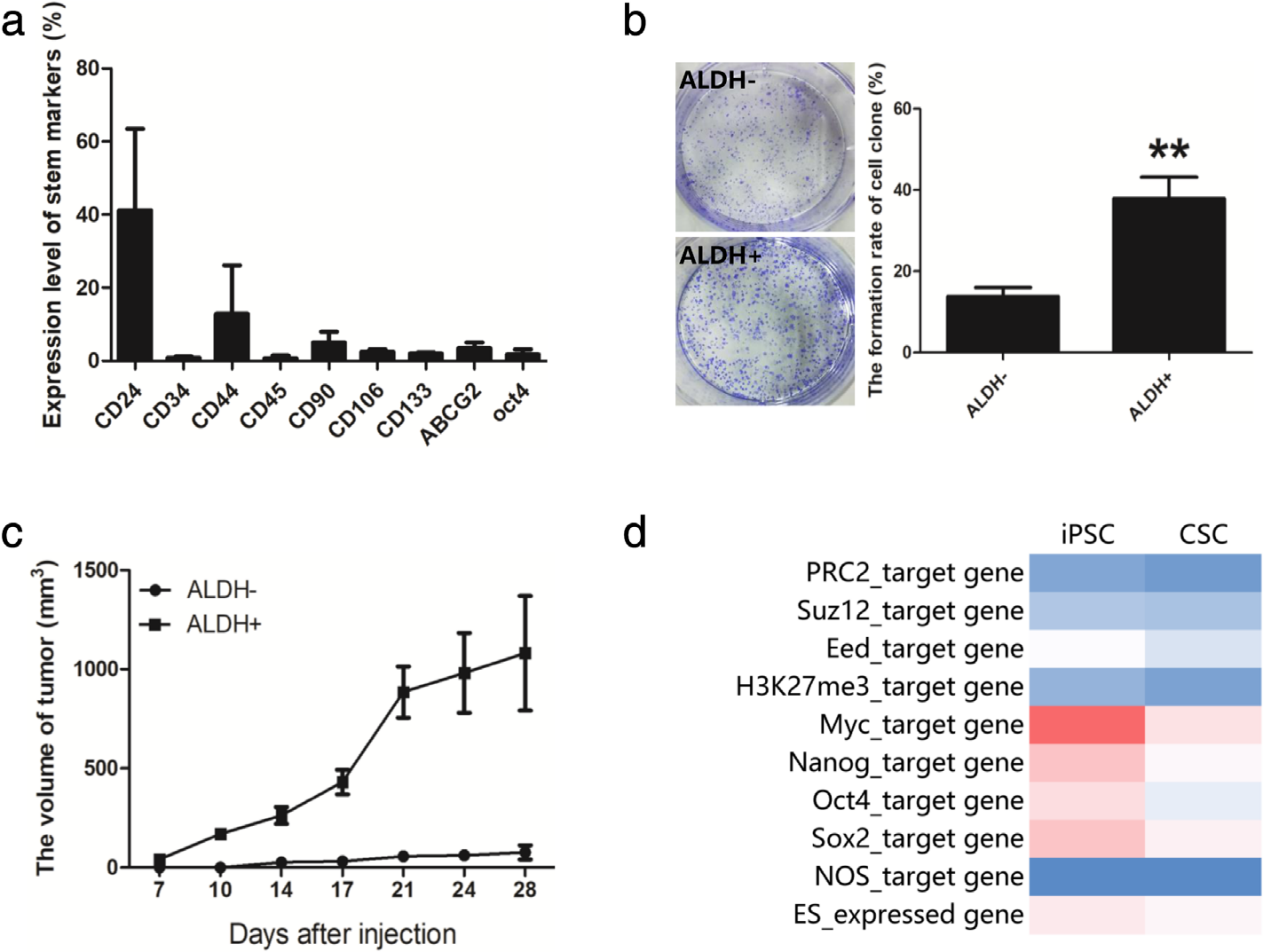
Identification of lung adenocarcinoma stem cells and its similarities to iPSC. Primary lung adenocarcinoma tissues were collected and digested into single cells and ALDH+ stem cells were sorted using flow cytometry (*n* = 3). (**a**) The stem cell markers were stained and analyzed using flow cytometry. (**b**) Clone formation ability of ALDH‐ and ALDH+ cells. (**c**) 10^3^ of ALDH‐ or ALDH+ cells subcutaneously injected and the tumor volume monitored. (**d**) Gene set enrichment in CSCs compared to iPSCs.

Human lung adenocarcinoma tissue was digested and successfully cultured using a primary tumor cell culture kit (IMMORTECH, China). ALDEFLOUR kit (Stem Cell, USA) was used to evaluate and isolate the ALDH positive cancer stem cells from the primary tumor cells (*n* = 3). Flow cytometry was used to analyze the expression of other stem cell markers. The findings revealed that ALDH, CD24, CD44, and CD90 were highly expressed in the isolated cancer stem cells (Fig 1a). In addition, the ability to form a clone and in vivo tumor formation were observed in ALDH+ cells (Fig 1b and c). All the ALDH+ cells from human lung adenocarcinoma tissues showed typical stem‐like characteristics and were chosen for sequencing analysis.

The gene group related through a common function or pathways was considered to be gene sets and the expression analyses of gene sets could prove more revealing than single‐gene analyses.^2^ A total of 10 gene sets were selected and were associated with stem characteristics including ES expressed genes,^28^, ^29^ Nanog, Oct4 and NOS targets,^30^ Myc targets^31^ and polycomb targets.^2^, ^32^ As shown in Fig 1d, a similar gene expression level was observed between CSC and iPSC.

### Humanized mice for tumor antibody production

**Figure 2:**
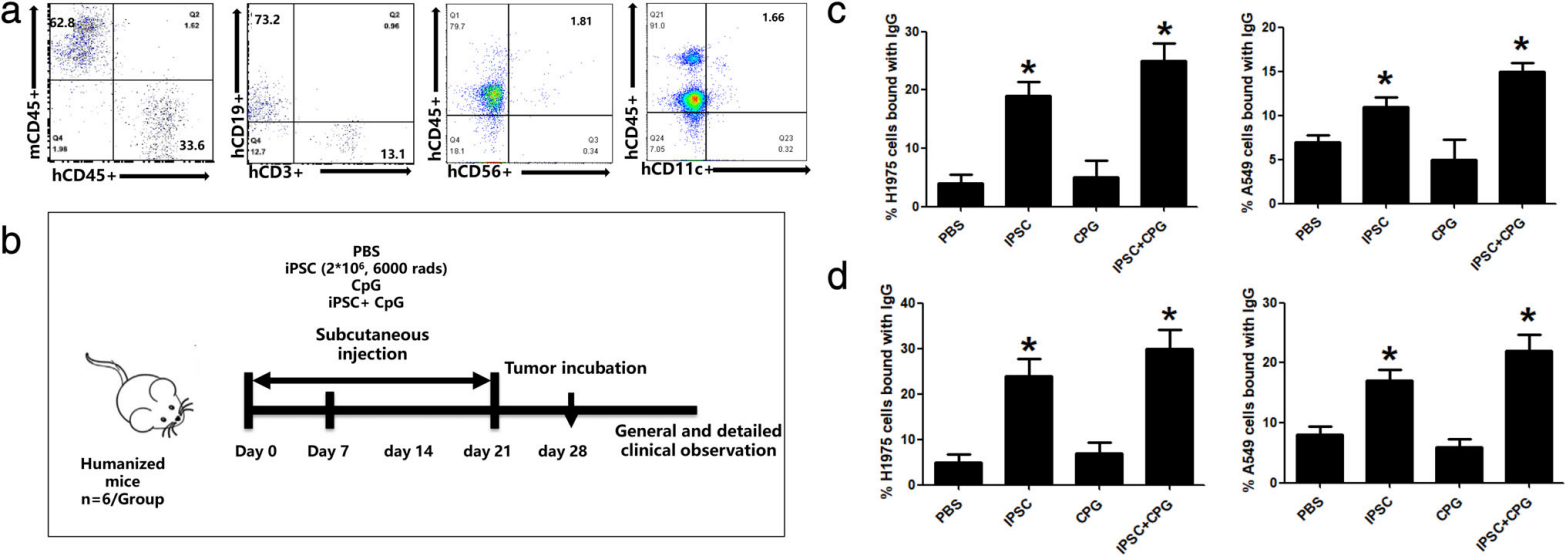
Human fibroblast‐derived iPSC elicits tumor‐specific antibody production in a humanized mouse model. (**a**) Humanization of the mice. (**b**) The brief procedure of immunization and sample collection. Representative FACS plot of serum IgG binding of PBS, iPSC, CPG and iPSC+CPG (**c**) two weeks and (**d**) four weeks after immunization. Statistical results are expressed as the means ± SE with *n* = 6 in each group. *, represents *P* < 0.05.

Humanize B‐NDG mouse models were established by engrafting human CD34^+^ umbilical cord stem cells. Fig 2a shows that more than 20% of human CD45+ cells were detected 12 weeks after injection. Mice with a lower percentage were eliminated. Human iPSC and CpG were used as a combined vaccine to elicit protective immune responses against tumors and PBS was used as a negative control. Fig 2b shows the experimental procedure. Serum samples were collected at two and four weeks after the first injection and the tumor binding IgG detected. As depicted in Fig 2c and d, iPSC and iPSC+CpG induced an increase in the production of A549 and H1975 specific IgG antibodies compared with the PBS group. Moreover, a higher IgG ratio was observed in week 4 compared with week 2.

### iPSC+CpG pretreated mouse exerts stronger immune cell responses against lung cancer

**Figure 3:**
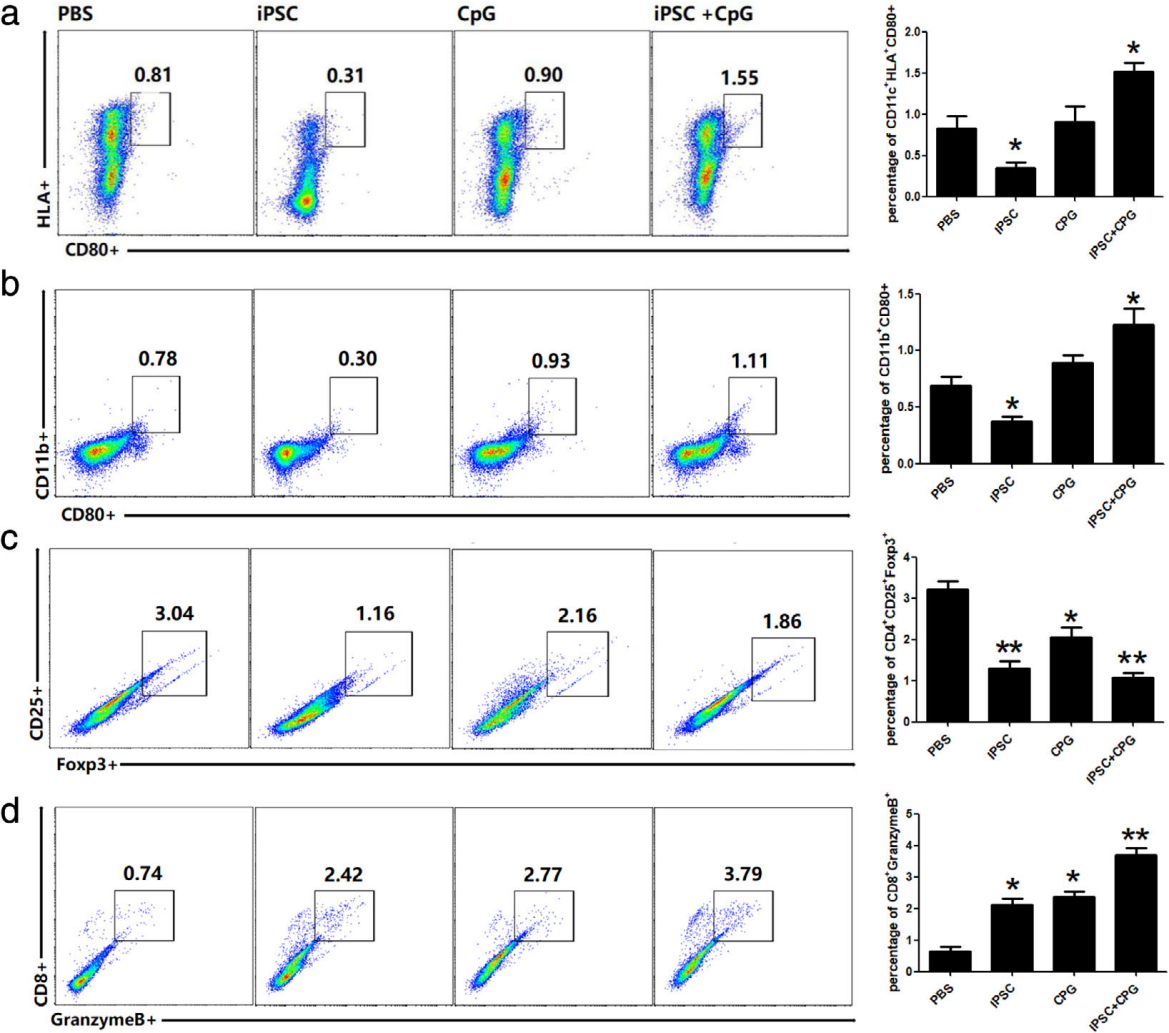
Preimmunization with hiPSC and CPG induce a higher percentage of antigen‐presenting and cytotoxic T cells in the spleen. Two weeks after A549 introduction, percentage of (**a** and **b**) antigen‐presenting cells;, (**c**) regulatory T cells; and (**d**) cytotoxic T cells in the spleen as analyzed using fluorescence‐activated cell sorting (FACS). Statistical results are expressed as the means ± SE with *n* = 6 in each group. *, represents *P* < 0.05.

Human lung cancer cell line A549 was introduced to the mice after pre‐immunization, to evaluate iPSC+CpG combined vaccine ability to induce an effective cellular immune response. This study revealed that after two weeks of tumor‐bearing, a higher percentage of spleen antigen‐presenting cells (CD11c^+^, HLA^+^, CD80^+^_,_ and CD11b^+^) was observed in the iPSC+CpG group (Fig 3a and b). Besides, a significant decrease in the Treg ratio was observed in the iPSC, CpG and iPSC+CpG group (Fig 3c) and a significant increase in the cytotoxic T cell ratio was observed in the three groups and more so in the iPSC+CpG group (Fig 3d).

### iPSC+CpG vaccination provides protective immunity against lung cancer

**Figure 4:**
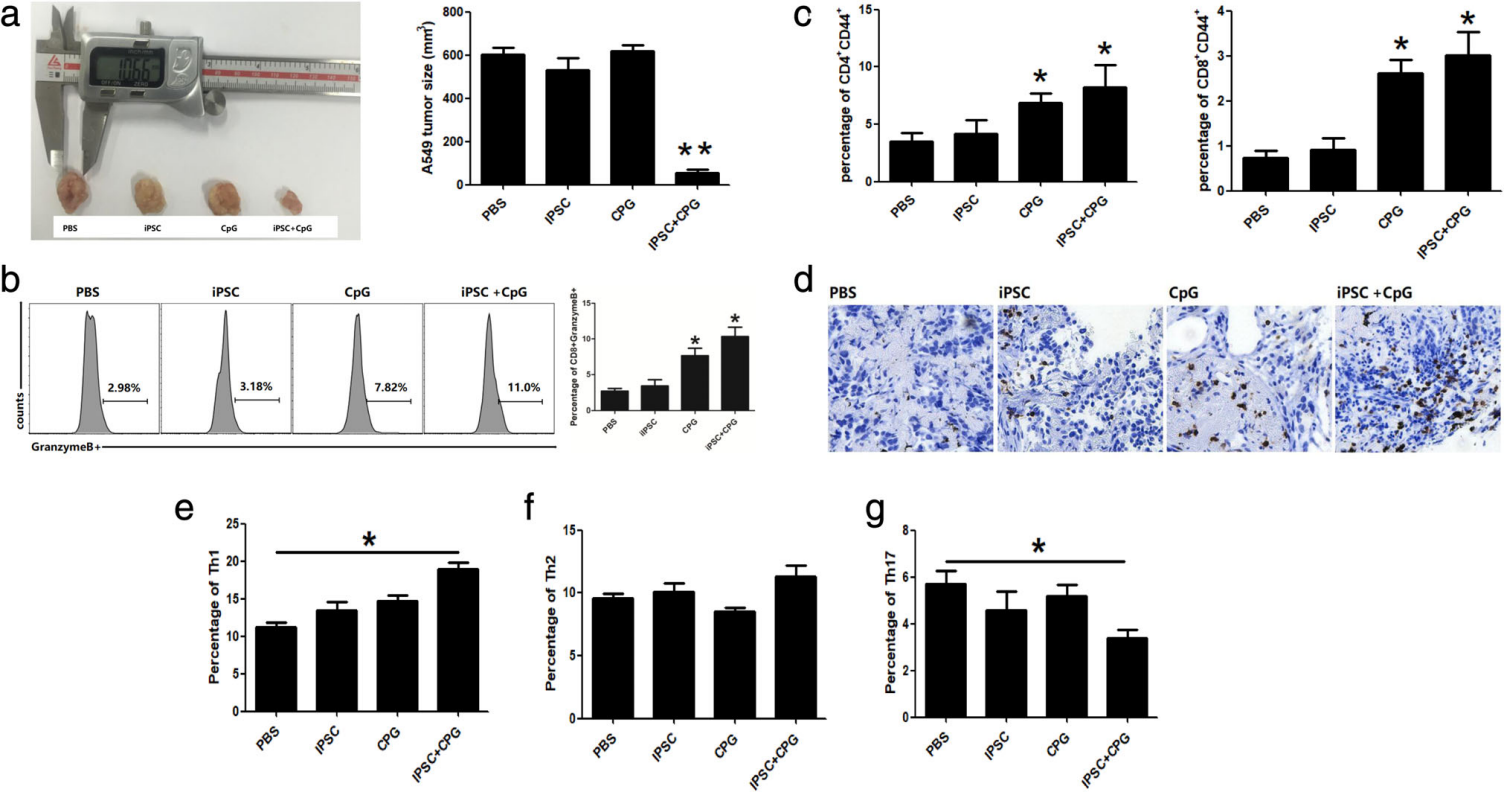
Preimmunization with hiPSC and CPG promotes antitumor responses and significantly inhibits tumor growth. (**a**) Representative tumor images of the hiPSC+CPG group compared with other groups. (**b**) Percentage of cytotoxic T cells in the spleen. (**c**) Percentage of T cell populations in draining lymph nodes. (**d**) CD8 + T cell infiltration in tumor tissues. Percentage of (**e**) Th1; (**f**) Th2; and (**g**) Th17 cells in the spleen. Statistical results are expressed as the means ± SE with *n* = 6 in each group. *, represents *P* < 0.05.

Tumor growth was monitored four weeks after tumor introduction. Tumor volume in the iPSC+CpG group was significantly reduced compared to that in the other groups (Fig 4a, Fig S1). The cytotoxic T cell subsets in the spleen were higher in CpG and iPSC+CpG groups than in the PBS group (Fig 4b). Increased effector/memory helper T cells (CD4 + CD44+) and effector/memory cytotoxic T cells in the peripheral blood were observed in the CpG and iPSC+CpG groups (Fig 4c). Higher CD8 + T cell infiltration in tumor tissues of the iPSC+CpG group was also observed (Fig 4d). A higher percentage of spleen Th1 cells was also observed in the iPSC+CpG group (Fig 4e) and the Treg ratio was reduced (Fig 4g). There was no significant difference in the Th2 ratio in all the groups (Fig 4f).

### Adoptive transfer of spleen T cells from iPSC+CpG preimmunized mouse reduces tumor volume

**Figure 5:**
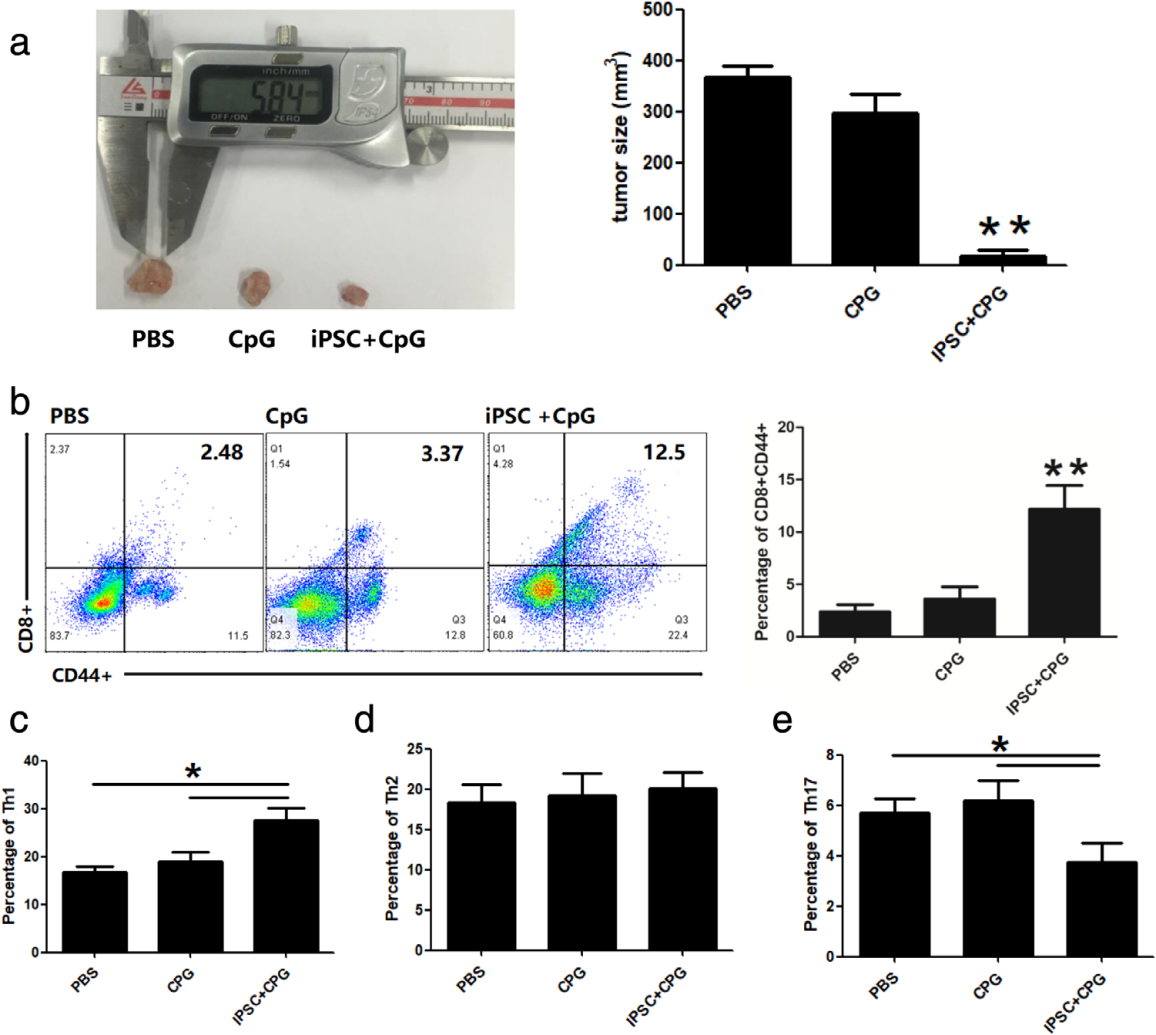
Spleen T cell transfer from iPSC+CPG preimmunized mouse inhibits tumor growth. After immunization with PBS, CPG, and iPSC+CPG, the mice were sacrificed and the spleen CD3 + T cells were isolated and injected intravenously into the tumor (*n* = 4/group). (**a**) Two weeks after T cell transfer, quantified tumor sizes; (**b**) cytotoxic T cells; (**c**) Th1 cells; (**d**) Th2 cells; and (**e**) Th17 cells in the spleen as analyzed using FACS. Statistical results are expressed as the means ± SE. *, represents *P* < 0.05.

Mice in all the groups were sacrificed and their spleen CD3 + T cells isolated for use in evaluating whether the raised cytotoxic T cells in the preimmunized groups could terminate tumor growth. As shown in Fig 5a and Fig S1, the tumor volume in the iPSC+CpG group was significantly reduced two weeks after T cell transfer compared with the PBS and CpG groups. In addition, the percentage of CD8 + CD44 + T cells was also increased in the iPSC+CpG group (Fig 5b). The spleen Th1 subset in the iPSC+CpG group was higher than that in the other two groups (Fig 5c), whereas there was no significant difference in the Th2 subset in all the three groups (Fig 5d). The Th17 ratio was reduced in the iPSC+CpG group (Fig 5e).

## Discussion

Immune system mobilization is important for tumor elimination.^33^ Since the tumor microenvironment negatively impacts the immune response, strategies aimed at reversing immunosuppression induced by tumors have been developed including immune checkpoint blockade and chimeric antigen receptor T cell therapy.^34^, ^35^, ^36^ However, the efficiency of these strategies varies from one person to the other and the immune cell‐based vaccine therapies are very expensive.^37^, ^38^ Because of this, there is a need to develop novel tumor therapy approaches that are efficient for cancer patients and also less costly.

Tumor‐associated antigen‐based vaccines provide protective immunity by inducing antigen‐specific immune responses.^39^ However, immunization with one or several antigens may fail to control tumor development. In addition, the isolation and identification of tumor‐specific antigens are challenging.^40^, ^41^ This, therefore, makes the use of the whole cell‐based vaccine another suitable and attractive option. Tabatabaei *et al*. demonstrated that vaccination using human amniotic epithelial cells induced effective protection against colon adenocarcinoma in a murine model.^19^ Other previous studies have also suggested that pluripotent cells share tumor‐specific antigens with cancer cells^17^ and may induce antitumor immunity in vivo.

Solid tumors are organized with a small number of CSCs which drive tumor growth, repopulation after therapy and metastasis.^42^ Strategies targeting CSCs can significantly delay relapse compared with other traditional cancer therapies.^43^ In this study, CSCs were found to share multiple characteristics with iPSCs. Therefore, iPSCs can serve as a choice for CSCs to simplify and shorten the cell culture process. In addition, preimmunization with the iPSC‐based vaccine was shown to produce an effective immune response in a humanized lung cancer mouse model. The A549 and H1975 specific serum IgG were reported to be significantly increased two weeks after the first injection, an indication of shared antigen between iPSC and tumor cells. Antigen‐presenting cell, helper T cell, and cytotoxic T cell were also reported to be systemically upregulated. Also, reduced levels of Th17 and Treg were reported. Considering the role of Treg and Th17 in tumorigenesis,^44^, ^45^, ^46^ this study demonstrates that the use of iPSC as a tumor vaccine is promising. The tumor volume of the iPSC+CpG preimmunized group was reported to be significantly reduced compared with the control group and the CD8 + T cell infiltration was also enhanced in this group. Adoptive transfer of spleen T cells in the iPSC+CpG preimmunized group led to an increase in the cytotoxic T cell ratio and a reduction in tumor volume. Kooreman *et al*. proved that autologous iPSC‐based vaccines elicit antitumor responses in murine breast cancer, mesothelioma and melanoma models.^11^ Research carried out by Li *et al*. showed that vaccination with human iPSC generates immune responses against mice CT26 colon carcinoma.^17^ Our results confirm the possibility of employing universal human induced pluripotent stem cells (hiPSCs) to elicit protective antitumor responses on humanized mice model.

This study assessed the preliminary effects of iPSC‐based tumor vaccine; however, it is important to note that differences exist between a humanized mouse model and human immunology.^47^ Therefore, further experiments are still needed to determine the efficiency of iPSC‐based vaccines on human samples to validate these findings. In addition, our findings revealed that iPSC‐based vaccine did not completely stop tumorigenesis as we observed tumor growth in all the three groups. This shows that future research needs to focus on establishing other strategies that can completely terminate tumor growth.

In conclusion, this study shows the possibility of developing a universal tumor vaccine using iPSC, since they share multiple antigens with tumor cells. In this study, preimmunization with human iPSC‐based vaccine‐induced functional cytotoxic T cell responses in both the spleen and tumor tissues. In addition, tumor growth was reduced and no side effects were observed. These findings will assist in exploring other new tumor therapeutic options in future research.

## Disclosure

The authors have no conflicts of interest to declare.

## Supporting information


**Figure S1** Tumor size of all experimental mice. (**a**) Tumor images of the hiPSC+CPG group compared with other groups after tumor introduction. (**b**) After immunized with PBS, CPG and iPSC+CPG four times, mice were sacrificed and spleen CD3 + T cells were isolated and injected intravenously into tumor incubation mice. Two weeks after T cell transfer, tumor sizes were quantified.
