# Supplementary material for: Expression levels of a gene signature in hiPSC associated with lung adenocarcinoma stem cells and its capability in eliciting specific antitumor immune‐response in a humanized mice model
Source: Thorac Cancer. 2020 Apr 20;11(6):1603–12. doi: 10.1111/1759-7714.13440 (PMC7262930; doi:10.1111/1759-7714.13440)
Supplement: Supplementary file 1 — Figure S1 Tumor size of all experimental mice. (a) Tumor images of the hiPSC+CPG group compared with other groups after tumor introduction. (b) After immunized with PBS, CPG and iPSC+CPG four times, mice were sacrificed and spleen CD3 + T cells were isolated and injected intravenously into tumor incubation mice. Two weeks after T cell transfer, tumor sizes were quantified. [file TCA-11-1603-s001.docx]

**Expression levels of a gene signature in hiPSC associated with lung adenocarcinoma stem cells and its capability in eliciting specific anti-tumor immune-response in a humanized mice model**

Jingbo Wang^1,2,3^, Lijuan Shao^1,2,3^, Liujing Wu^1,3^, Wei Ma^1,3^, Yuanyuan Zheng^1,3^, Chaofeng Hu^3*^ and Furong Li^1,2,3*^

^1^ Translational Medicine Collaborative Innovation Center, The Second Clinical Medical College (Shenzhen People’s Hospital), Jinan University, Shenzhen 518020, China.

^2^ Shenzhen key laboratory of stem cell research and clinical transformation, Shenzhen 518020, China

^3^ Integrated Chinese and Western Medicine Postdoctoral research station, Jinan University, Guangzhou 510632, China


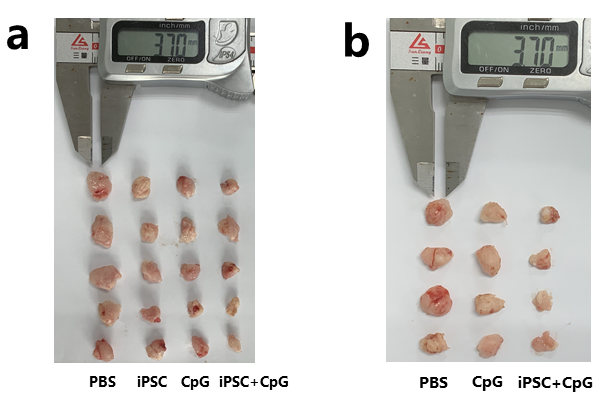


**Figure S1. Tumor size of all experimental mice.** (a) Tumor images of hiPSC+CPG group compared with other groups after tumor introduction. (b) After immunized with PBS, CPG and iPSC+CPG 4 times, mice were sacrificed and spleen CD3+T cells were isolated and injected intravenously into tumor incubation mice. 2 weeks after T cell transfer, tumor sizes were quantified.
